# Stellate cells and mesenchymal stem cells in benign mammary stroma are associated with risk factors for breast cancer – an observational study

**DOI:** 10.1186/s12885-018-4151-x

**Published:** 2018-02-27

**Authors:** Björn Logi Isfoss, Bo Holmqvist, Elin Sand, Johan Forsell, Helena Jernström, Håkan Olsson

**Affiliations:** 1 Department of Clinical Sciences, Lund, Division of Oncology and Pathology, Lund University, Lund, Sweden; 2 Department of Pathology, Skane University Hospital, Lund, Sweden; 3 Department of Pathology, Telemark Hospital, Ulefossv. 55, 3710 Skien, Norway; 4 ImaGene-iT, Medicon Village, Lund, Sweden; 5 Department of Clinical Sciences, Lund, Division of Cancer Epidemiology, Lund University, Lund, Sweden

**Keywords:** Breast, Breast cancer, Cancer risk, Familial cancer, Stem cells, Histology, Aldehyde dehydrogenase, BRCA1, BRCA2, Immunohistochemistry

## Abstract

**Background:**

It is not known whether stromal cells in benign breast tissue can mediate risk of breast cancer. We recently described aldehyde dehydrogenase 1 A1 (ALDH1) positive (+) cells in morphologically normal breast stroma of premenopausal women, and the data indicated that their distribution is associated with clinical risk factors for breast cancer. The aim of the present study was to define the identities of these cells using histologic and immunohistologic methods, and to investigate associations between those cells and hormonal and genetic risk factors in pre- and postmenopausal women.

**Methods:**

Stroma of morphologically normal tissue was analyzed in samples from 101 well-characterized women whose breasts had been operated. Morphology and immunolabeling were applied to determine cell identities based on the putative stem cell markers ALDH1 and stage-specific embryonic antigen-3 (SSEA3), and immunophenotypes indicating mast cells or stellate cells. The results were compared with the patients’ risk factors using regression analysis (two-tailed).

**Results:**

ALDH1+ round/oval cells were associated with low parity in BRCA1/2 carriers (*p* = 0.022), while in non-BRCA1/2-carriers they were negatively associated with nulliparity (*p* = 0.057). In premenopausal women ALDH1+ round/oval cells were associated with family history (*p* = 0.058). SSEA3+ round/oval cells were morphologically and immunohistologically consistent with multilineage stress-enduring (Muse) cells, and these cells were independently associated with the breast cancer risk factors low parity (*p* = 0.015), family history (*p* = 0.021), and hormone use after menopause (*p* = 0.032). ALDH1+ spindle-shaped/polygonal cells were immunohistologically consistent with stellate cells, and were negatively associated with family history of breast cancer (*p* = 0.001).

**Conclusion:**

This study identified novel stromal cell types in benign breast tissue that have a potential for stratifying women for breast cancer risk.

**Electronic supplementary material:**

The online version of this article (10.1186/s12885-018-4151-x) contains supplementary material, which is available to authorized users.

## Background

Nulli- and oligoparous women are at increased risk of developing breast cancer [1–3]. The risk is also higher for postmenopausal women who have received hormone therapy [4–7] and is probably at a similar level for those using the contraceptive pill [8–10]. Hereditary factors account for 5–10% of breast cancers, of which only 20–25% are attributed to known genes such as *BRCA1*, *BRCA2*, and *TP53* [11]. It is has not been determined whether specific cell types in benign breast stroma are associated with susceptibility to breast cancer. The aim of the present study was to identify stromal cells in benign breast tissue and ascertain whether these cells are mediators of risk.

Most studies of cells in relation to mammary oncogenesis have focused on epithelial cells, whereas the importance of stromal stem cells is poorly understood. Also, the majority of oncogenesis-related studies of breast tissue have been performed on mechanically or chemically dissociated cells and thus have had no histological reference. Furthermore, in light of the beneficial effects of early cancer diagnosis, it might be advantageous to screen healthy women for the risk of breast cancer by performing core biopsies, a type of test that could be based on immunohistologic identification of specific epithelial or stromal cells. For these reasons, we conducted the present study to elucidate the identities of different types of stromal cells in histologically normal female breast tissue, and also to determine whether those cells are associated with clinical risk factors for breast cancer. We hypothesized that the population of round or oval-shaped (r/o) aldehyde dehydrogenase 1 A1 positive (ALDH1+) cells in the stroma of terminal duct-lobular units (TDLUs) includes mesenchymal stem cells, and that the population of ALDH1+ spindle-shaped or polygonal (s/p) cells in the same location includes stellate cells.

Considering that anti-cancer therapy is now being designed to target stem cells [12], it is essential to map the normal histological distribution of stem marker-positive cells. Both benign stem cells and cancer stem cells in breast tissue have been reported to express ALDH1 [13, 14]. ALDH1 is a member of an enzyme family that contributes to maintaining cells intact via the detoxification of aldehydes [15], promotes cell differentiation, and converts vitamin A to its physiologically active form retinoic acid [16]. Previous studies have indicated that ALDH1 protein expression is scarce in stroma of breast carcinoma, and when present it is associated with favorable patient survival [17, 18].

In our earlier investigations of benign female breast tissue we used morphological and immunohistochemical methods to demonstrate that ALDH1+ cells are ductal, ductular, or stromal, and have no detectable proliferative activity [14], and also found that such cells are associated with established risk factors for breast cancer [19, 20]. Two types of ALDH1+ stromal cells were morphologically identified, which we designated r/o cells and s/p cells. Correlating those cells with breast cancer risk factors specifically in premenopausal women showed that having a low number of ALDH1+ CD44+ CD24– r/o cells in the stroma of TDLUs was associated with family history of breast cancer, and having a low number of ALDH1+ CD44– CD24– s/p cells was associated with the breast cancer risk factor nulliparity [20]. Conversely, a *high* number of ALDH1+ cells in ductular epithelium was associated with the same risk factors and also with genetic risk factors for breast cancer [19]. These findings indicate that different types of stromal ALDH1+ cell types play disparate roles, and also suggest that the histological location and cell-specific identity of these and other immunophenotypic cells can provide an important basis for an improved assessment of the risks of developing breast cancer.

The morphologies the ALDH1+ r/o and s/p stromal cells that we recently described in benign breast tissue correspond morphologically to mast cells and mesenchymal stem cells [21, 22], and fibroblasts/fibrocytes and stellate cells [23–26], respectively. The last mentioned cell type is present in a wide range of tissues, and is also called the vitamin A-storing cell [23]. In previous studies [14, 20], we described numerous ALDH1+ s/p cells in TDLU stroma that are thin and elongated in shape and display in some tissue planes triangular cell bodies. We also demonstrated that ALDH1+ s/p cells are negative for the following: the muscle protein marker smooth muscle myosin heavy chain (SMMHC), the proliferation marker Ki-67, the pan-leukocyte marker CD45, and the epithelial cell markers Cam5.2 and E-cadherin [14]. Furthermore, the morphology and high ALDH1 expression of ALDH1+ s/p cells, along with the ubiquitous presence of these cells in subepithelial locations, suggest that they correspond to stellate cells. Stellate cells contain substantial amounts of ALDH1, an enzyme that is critical for the synthesis of retinoic acid [16].

These observations prompted us to identify ALDH1+ stromal cells that are associated with risk factors for breast cancer. To this end we applied in situ protein-detecting methods using markers for stemness and cell differentiation in morphologically normal breast tissue. Quantitative data were then compared with data from patient records concerning the presence or absence of specific hormonal and genetic breast cancer risk factors.

The cell types described here are novel for breast tissue, and if the results are validated in an independent cohort they may provide a basis for a future biopsy-based test that can stratify women with regard to the risk of breast cancer.

## Methods

The study was done by applying histoloic and immunohistologic methods on benign tissue from women that were selected into the study for having been operated for benign and malignant conditions of the breast. The findings were compared with clinical data for examining possible assocations between cell types and risk factors for breast cancer.

### Clinical material

**Fig. 1:**
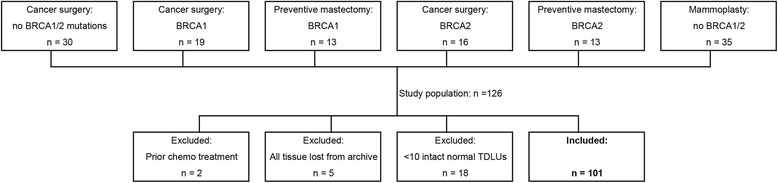
Patients contributing the histologically normal breast tissue samples analyzed in the present study. Patient groups considered for inclusion in the investigation were selected based on four different breast surgery indications: cancer but no *BRCA1/2* mutation; cancer and *BRCA1/2* mutation; *BRCA1/2* carrier status but no cancer; mammoplasty. The same set of patients was evaluated in previous studies [14, 19, 20]

The tissue material used in this investigation consisted of formalin-fixed and paraffin-embedded (FFPE) breast specimens from the patient groups shown in Fig. 1. Only histologically benign tissue that was free from epithelial atypia or hyperplasia was studied. The tissue samples were obtained during breast operations performed on women who had received care for breast cancer or for *BRCA1/2* carrier status, or had undergone reduction plastic surgery, at Skåne University Hospitals (Lund, Malmö, Helsingborg, Kristianstad) during the period 1983 to 2010. Clinical and immunohistologic data on the same set of patients have been used in a previous study [19].

One hundred twenty-six patients belonging to different clinical categories were randomly selected: 30 with breast cancer, 61 diagnosed with a deleterious *BRCA1* or *BRCA2* mutation, and 35 who had undergone reduction mammoplasty (members of a previously reported cohort [27]). Patients were excluded as follows: two due to prior neoadjuvant therapy; five because all tissue was lost from archive; 18 because only a limited number of histologically normal TDLUs (< 10) were present in any one tissue block by means of microscopy of the original slides stained with hematoxylin and eosin (H&E). The tissue block from each patient that contained the largest number of histologically normal glandular breast tissue was used in the analyses. For each of the selected patients, the median area of tissue section that was finally analyzed was 255 (range 48–621) sq. mm.

The 101 patients who were found eligible for tissue analysis had been diagnosed as follows: 49 with no breast cancer, four with in situ carcinoma (ductal and/or lobular), 40 with invasive ductal carcinoma, seven with invasive lobular carcinoma, and one with mixed invasive ductal and lobular carcinoma. The median age of these patients was 39 years (range 20–81 years). Sixty-two women were premenopausal at the time of surgery, and 32 were postmenopausal. The menopausal status of seven women was not known. Thirty-seven women were nulliparous, 58 were parous, and six had unknown parity. Sixty patients had a first-degree relative with breast cancer, and 27 of those subjects had a *BRCA1* mutation and 22 a *BRCA2* mutation. One patient had unknown family history and no confirmed *BRCA1/2* mutation. The investigators were blinded for the clinical information while analyzing specimens in the laboratory.

All samples from breast operation specimens were initially evaluated using the original H&E-stained sections. In addition to the criteria mentioned above, patient material was chosen from tissue blocks that contained the largest amount of histologically normal glandular tissue including ≥10 benign TDLUs.

A limited number of randomly selected samples (*n* = 16) were used for the initial identification of specific cell types (double immunofluorescence) present in the study population, with no comparisons between patient groups. Then a total of 101 patients were used for statistic evaluations of the identified specific cell types (immunohistochemistry), with comparisons between the patient groups.

### Double immunofluorescence labeling

The presence and identity of r/o and s/p cell types was initially performed by means of double immunofluorescence (dIF) labeling. An antibody against ALDH1 was used together with antibodies described as markers for mesenchymal stem cells, mast cells, and stellate cells: ALDH1 together with SSEA3, ALDH1 with tryptase, tryptase with SSEA3, and ALDH1 with vinculin. Archival FFPE samples of tissue types recommended by the manufacturers were used to ensure specific labeling of the cell types that were targeted in this study. The dIF labeling was evaluated as cellular presence and/or co-presence of the antigens. This qualitative analysis was performed on representative samples to enable morphologic identification of specific cell types expressing one or more of the investigated antigens (i.e. ALDH1, SSEA3, tryptase, and vinculin). For the double immunofluorescence labeling, 16 premenopausal women of age 31–40 years were randomly selected from the patient groups with the following breast cancer risk categories (see Table 1): nulliparous (*n* = 3), parous (*n* = 3), family history of breast cancer but no *BRCA1/2* mutation (*n* = 4), *BRCA1* mutation (*n* = 3), and *BRCA2* mutation (*n* = 3).

**Table 1:** Patient characteristics for the subset that was selected for double immunofluorescence experiments; all of age 31–40 years

Patient	Child birth(s)	Family history of breast cancer	*BRCA1/2* mutation	Pathology diagnosis
17	No	No	No	IDC
75	No	No	No	IDC
91	No	No	No	Benign
62	Yes	No	No	Benign
97	Yes	No	No	IDC
59	Yes	No	No	Benign
89	Yes	Yes	No	IDC
78	No	Yes	No	IDC
30	No	Yes	No	IDC
83	Yes	Yes	No	IDC
46	Yes	Yes	*BRCA1*	DCIS
99	Yes	Yes	*BRCA1*	IDC
103	Yes	Yes	*BRCA1*	IDC
116	No	Yes	*BRCA2*	IDC
37	Yes	Yes	*BRCA2*	Benign
126	Yes	Yes	*BRCA2*	Benign

*Abbreviations: IDC* invasive ductal carcinoma *DCIS *ductal carcinoma in situ

For double immunofluorescence labeling, paraffin sections were heated at 60 °C for 30 min and then deparaffinized, starting in xylene followed by hydration in a graded alcohol series, ending with water. Thereafter, the slides were immersed in citrate acid buffer (10 mM, pH 6) containing 0.05% Tween 20 (both from Sigma-Aldrich, St. Louis, MO, USA). Heat-induced antigen retrieval was performed in a microwave oven (3 min at 800 W to 95 °C, followed by 10 min at 200 W and 82 °C). The slides were then allowed to cool to RT before being immersed in distilled water (at RT) and subsequently rinsed in phosphate-buffered saline (PBS, pH 7.4) (Medicago, Uppsala, Sweden).

The dIF immunolabeling started with incubation of sections for 30 min at RT in blocking solution composed of PBS containing 1% BSA (Sigma-Aldrich) and 0.05% Triton X100 (Applichem). Next, the sections were incubated for 16 h at 4 °C in a mixture of two primary antibodies raised against various antigens in different animal host species (see Table 2). Thereafter the sections were rinsed in PBS and incubated for 30 min at RT in a mixture of secondary antibodies made against the host species for the primary antibodies and conjugated with fluorophores for detection of the binding at different wavelengths. All antibodies were diluted in the blocking solution, and all incubations were performed in a moisture chamber. To ensure specific immunolabeling with the primary antibodies, and to exclude the risk of detecting unspecific secondary antibody binding and autofluorescence during analyses, primary antibodies were excluded from the labeling protocol for adjacent sections. Following rinses in PBS, nuclear staining was performed by incubating sections in 4′, 6-diamidino-2-phenylindole (DAPI, 0.05 μM, Invitrogen-Thermo Fisher Scientific, Waltham, MA, USA) for 15 min at RT. Sections were then mounted in Fluoroshield mounting medium (Abcam, Cambridge, UK).

**Table 2:** Primary antibodies used for simultaneous visualization of two antigens by double immunofluorescence or single immunohistochemical labeling of individual antigens

Epitope	Host species	Product code	Dilution IHC	Dilution dIF	Manufacturer
ALDH1 A1	Mouse (IgG)	611,194	1:1000	1:100	BD
SSEA-3	Rat (IgM)	MC-631	1:100	1:50	DSHB
Tryptase	Rabbit (IgG)	Ab134931	1:2000	1:2000	Abcam
Vinculin	Rabbit (IgG)	SAB4503069		1:300	Sigma-Aldrich

MC-631 (SSEA-3) was deposited to the DSHB by Solter, D. / Knowles, B.B

*Abbreviations*: *dIF* double immunofluorescence, *IHC* immunohistochemical labeling, *BD* Becton Dickinson Bioscience (Franklin Lakes, NJ, USA), *DSHB* Developmental Studies Hybridoma Bank (University of Iowa, Iowa City, IA, USA); Abcam (Cambridge, UK); Sigma-Aldrich (St. Louis, MO, USA)

### Analyses of double immunofluorescence labeling

To evaluate the cellular and subcellular localization of labeling by one or two primary antibodies, and to ensure primary antibody specificity and general quality of double labeling, selected dIF-labeled samples were analyzed by confocal laser scanning microscopy (Zeiss LSM710). X–Y scanning and Z-stacking (200–400-nm optical sections) were used to determine cellular co-localization of dIF labeling, or absence thereof, in morphologically defined r/o and s/p cells. Control sections without primary antibody showed no detectable fluorophores and no or only a very low background signal, which confirmed supporting the specific binding of the primary and secondary antibodies that were used. See Additional files 1, 2, 3, 4 and 5.

All the selected tissue material from the dIF-labeled sections from 16 patients was assessed in an epifluorescence microscope (Olympus IX73, Tokyo, Japan). Sections that contained at least 10 TDLUs of good histological quality were considered evaluable. The cellular immunofluorescence labeling was evaluated by recording the visual inspection performed while switching between channels and by analyzing digital images (grabbed with a Olympus DP70 b/w detector) including color-coded and merged overlays of the different channels (Cell Sense software, Olympus). The use of thin sections (5 μm) and the solitary appearance of stromal r/o and s/p cells made it possible to evaluate the morphologically identified r/o and s/p single cells as single or double labeled in a specific focal plane. Two of the authors (BLI and ES) conducted the dIF analyses for ALDH1 and SSEA3, ALDH1 and tryptase, tryptase and SSEA3, and ALDH1 and vinculin.

### Immunohistochemical labeling

The results of the dIF experiments (see above) further supported that stromal ALDH1+ r/o cells are morphologically consistent with mesenchymal stem cells (SSEA3+) and/or mast cells (tryptase+), and that some ALDH1+ s/p cells are morphologically consistent with stellate cells (ALDH1+ and vinculin+). Therefore, to enable statistic comparison with risk factors for breast cancer we performed IHC labeling of these stromal r/o and s/p immunophenotypic cells in the entire cohort (*n* = 101).

In the protocol used for the single IHC labeling, the sections (5 μm) were initially heated to 60 °C for 30 min and then deparaffinized and hydrated, and processed for heat-induced antigen retrieval (as outlined above). Sections were then quenched for endogenous peroxidase activity through incubation in 0.03% H_2_O_2_ (10 min at RT) and blocked for unspecific binding sites for the secondary antibodies by incubation in blocking solution (PBS containing 1% BSA and 0.05% Triton X100; 30 min at RT). Thereafter the sections were incubated with one primary antibody diluted in the blocking solutions for 16 h at 4 °C (see Table 2) and then rinsed in PBS and incubated with horseradish peroxidase (HRP)-conjugated secondary antibodies (listed in Table 3) diluted in blocking solution for 30 min at RT. Sections were subsequently rinsed in PBS, and the HRP conjugate was reacted for 10 min at RT in a PBS solution containing 0.5 mg/ml 3,3′-diaminobenzidine (DAB) and 0.05% H_2_O_2_. The sections were then counterstained in Mayer’s hematoxylin (Histolab) for 30 s, dehydrated in a graded alcohol series ending with xylene, and mounted and coverslipped in Pertex (Histolab).

**Table 3:** Secondary antibodies used for double immunofluorescence labeling or immunohistochemistry

Secondary antibodies for dIF	Product code	Visualization	Dilution	Manufacturer
Donkey anti-mouse IgG (Alexa Fluor 488 conjugated)	715–546-150	ALDH1	1:200	JIR
Goat anti-rat IgM (Rhodamine-Red conjugated)	112–295-020	SSEA-3	1:200	JIR
Donkey anti-rabbit IgG (Rhodamine red conjugated)	711–296-152	Tryptase, Vinculin	1:200	JIR
Goat anti-rabbit IgG Alexa Fluor 488 conjugated	A11070	Tryptase	1:200	Invitrogen
Secondary antibodies for IHC	Product code	Visualization	Dilution	Manufacturer
Goat anti-mouse IgG (HRP conjugated)	K4007	ALDH1	Pre-diluted	Dako/Agilent
Goat anti-rabbit IgG (HRP conjugated)	K4011	Tryptase	Pre-diluted	Dako/Agilent
Goat anti-rat IgM (HRP conjugated)	112–036-075	SSEA-3	1:100	JIR

*Abbreviations*: dIF double immunofluorescence, *IHC* immunohistochemical labeling, *JIR* Jackson ImmunoResearch (West Grove, PA, USA); Invitrogen, Invitrogen, Thermo Fisher Scientific (Waltham, MA, USA); Dako/Agilent, Dako, Agilent Technologies (Santa Clara, CA, USA)

Antibody controls were made in all IHC labeling experiments by excluding incubation of the primary antibodies from the protocol on adjacent parallel sections. The specific labeling of the primary antibodies that were used was confirmed by the lack of or no comparable labeling in these control sections, which thereby further supported the specific binding of the antibodies also for IHC labeling techniques (see immunofluorescence labeling above).

### Analyses of Immunohistochemical labeling

The IHC labeling was evaluated by visual inspection using microscopes equipped for bright-field microscopy (Zeiss Axioskop and Olympus IX73). The immunolabeling was assessed for individual patients by analyzing 10–40 histologically normal TDLUs. Immunolabeled r/o and s/p cells were recorded as present or absent, and whether they were located in TDLUs or in the border between TDLU stroma and generic stroma. The analyses were performed by two of the authors who are experienced in histology and histopathology (JF & BLI).

The assessment of ratios for TDLUs containing any r/o and s/p immunolabeled cells (ALDH1, SSEA3, or tryptase immunoreactive) were statistically correlated with risk factors for breast cancer according to the patients’ clinical records.

### Statistic analysis

The size of the studied material was not based on statistical power estimates. Correlations between identified immunophenotypes of cells, determined by microscope evaluations of dIF and IHC labeling, were tested by two-tailed Spearman’s rho. The multivariate analysis using regression (logistic for discrete variables and linear for continuous variables) included the breast cancer risk factors age, family history, and parity, and additional models also included ongoing use of oral contraceptives or hormonal replacement therapy (HRT). *P* < 0.05 was considered significant. All analyses were performed using IBM SPSS v. 20 software.

## Results

### Cell-specific characterization of stromal cells by immunofluorescence labeling

**Fig. 2:**
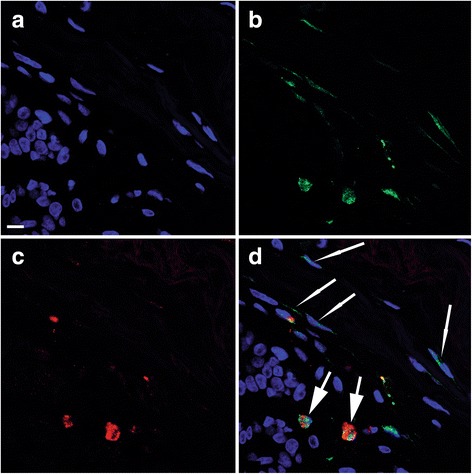
Confocal images of double immunofluorescence labeling of ALDH1 and SSEA3. Scale bars in all images 10 μm. **a–c** Three images of the same tissue area illustrating nuclear organization and morphology: DAPI labeling blue in **a**, ALDH1 labeling green in **b**, and SSEA3 labeling red in **c**. **d** A digital composite of the three images of ALDH1 and SSEA3 immunolabeling and DAPI staining. Two double-positive ALDH1+ SSEA3+ r/o cells (thick arrows) and four single-positive ALDH1+ s/p cells (thin arrows) are indicated

In TDLU stroma, r/o cells with ALDH1, tryptase, or SSEA3 immunolabeling exhibited morphology corresponding to that of mast cells. The results of dIF experiments using the various combinations of antibodies targeting SSEA3, ALDH1, and tryptase showed that at least one TDLU in specimens from all the investigated patients contained r/o cells displaying positivity for each of these antibodies. Double-labeled SSEA3+ ALDH1+ cells were detected in 13 of 16 patients (81%; see Fig. 2). SSEA3+ ALDH1– cells were present in 14 of 16 patients (87%) and SSEA3– ALDH1+ cells in 11 out of 16 patients (69%).

**Fig. 3:**
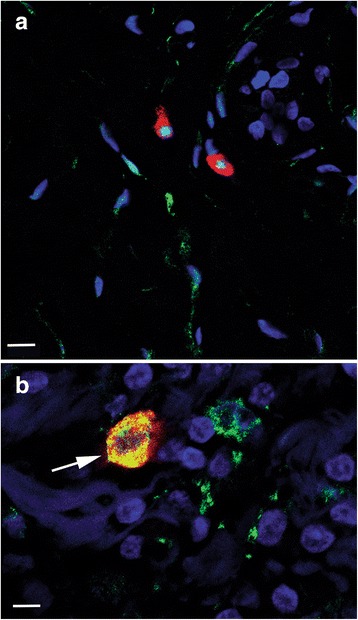
Confocal images of double immunofluorescence labeling of ALDH1 and tryptase. The images are digital composites from one focal plane showing merged channels with dIF-labeled ALDH1 (green) and tryptase (red), together with nuclear staining with DAPI (blue). **a** Image illustrating two ALDH1– SSEA+ round/oval cells (strong red) above scattered ALDH1+ SSEA– spindle-shaped cells (green). Scale bar 10 μm. **b** Image showing an area of tissue containing one large ALDH1+ tryptase+ double-labeled r/o cell (arrow). The ALDH1 labeling (green) to the right of this cell represents parts of ALDH1+ cytoplasm from different cell types. Double labeling appears as yellow. Scale bar 5 μm

ALDH1– tryptase+ r/o cells were observed in TDLU stroma of all patients, and ALDH1+ tryptase+ r/o cells were present in TDLUs of 15 patients (94%). ALDH1+ tryptase– r/o cells were detected in TDLUs of only nine patients (56%) (see Fig. 3).

**Fig. 4:**
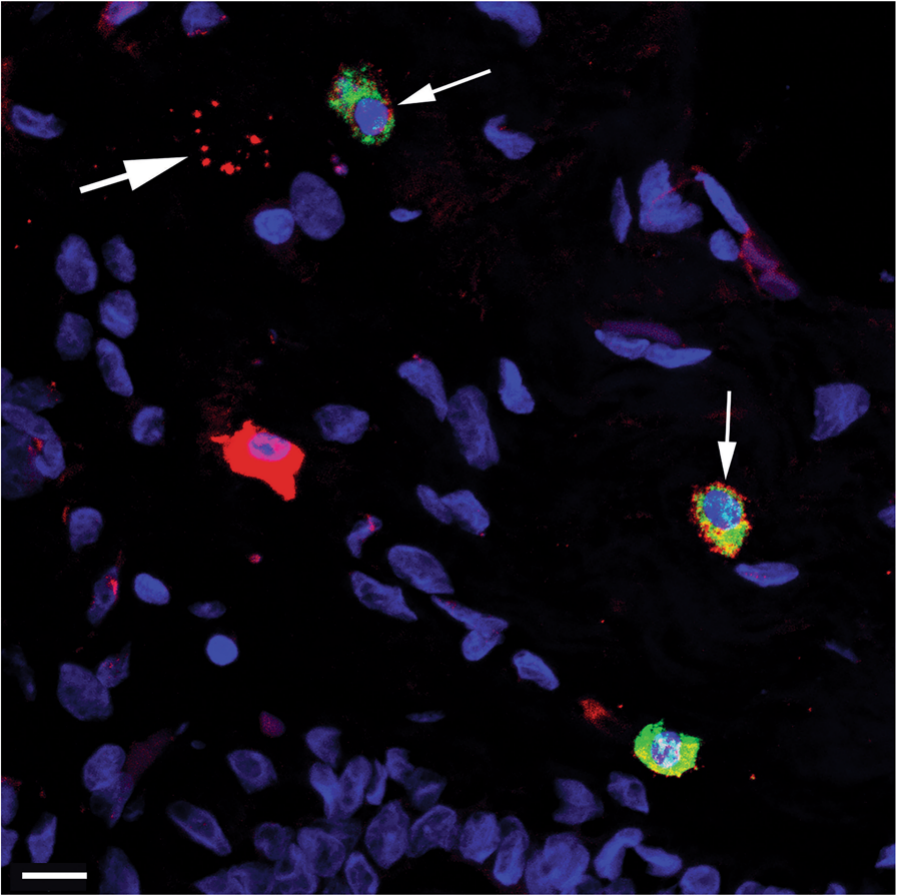
A confocal image of double immunofluorescence labeling of tryptase and SSEA3. The image is a “maximum intensity” digital composite from 12 focal planes showing merged channels from dIF labeling of tryptase (green) and SSEA3 (red), together with DAPI nuclear staining (blue). Double-labeled tryptase+ SSEA3+ round/oval cells (thin arrows) and a SSEA3+ degranulated cell (thick arrow) are indicated. Scale bar 10 μm

SSEA3+ and tryptase+ double-labeled r/o cells, as well as tryptase+ SSEA3– r/o cells, were found in 15 patients (95%). The same number of patients had tryptase– SSEA3+ cells. Interestingly, some of the r/o tryptase+ SSEA3– and tryptase+ SSEA3+ cells exhibited extracellular immunoreactive granules, which indicates degranulation **(**a phenomenon commonly associated with mast cells) (Fig. 4).

**Fig. 5:**
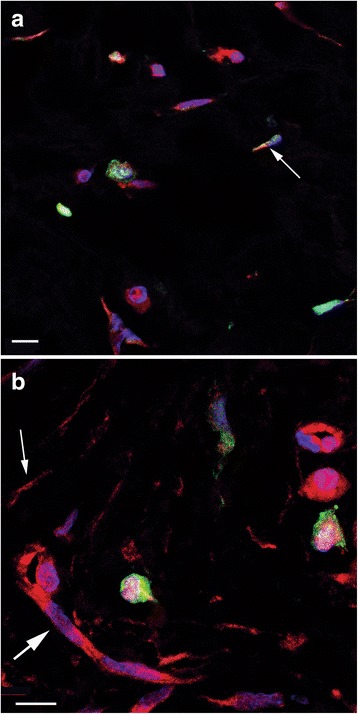
Confocal images of double immunofluorescence labeling of ALDH1 and vinculin. (**a** and **b**) The two images illustrate several spindle-shaped and polygonal ALDH1+ (green) and/or vinculin+ (red) cells, and the variable ALDH1 and vinculin immunolabeling of these cells. Nuclei are DAPI stained (blue). A thin arrow indicates an ALDH1 and vinculin double-labeled cell (yellow) in **a** and an ALDH– vinculin+ cell in **b**. The thick arrow in **b** indicates a capillary with vinculin+ endothelium (as reported previously [37]). Scale bars 10 μm

**Fig. 6:**
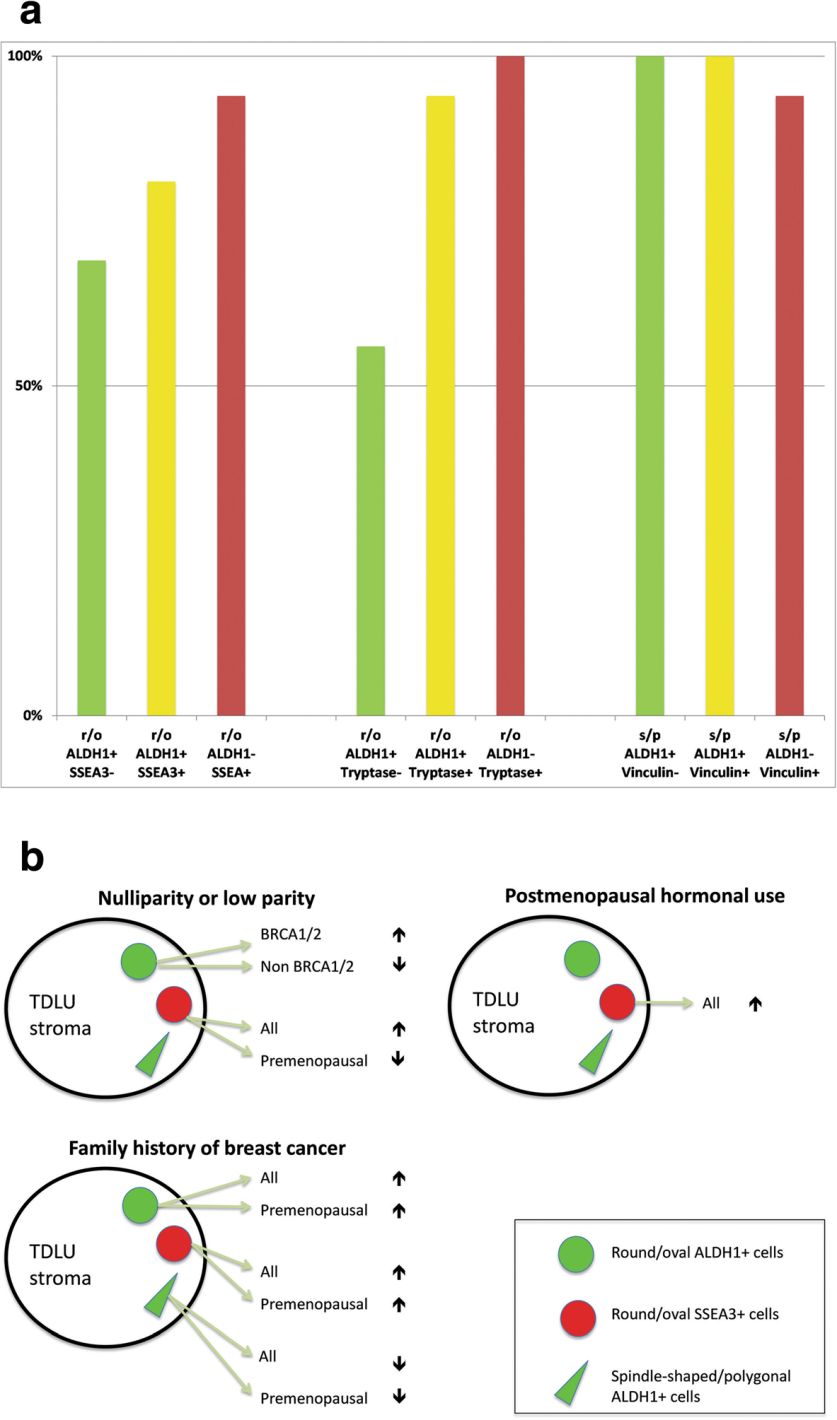
**a** Identified stromal cell immunophenotypes detected with double immunofluorescence labeling in histologically normal TDLUs. The result is presented as the percentage of patients (Y-axis) whose specimens contained one or more r/o and s/p cells that were double labeled (yellow) and/or single labeled with SSEA3 or tryptase (both red), or ALDH1 (green) in histologically normal TDLU stroma (*n* = 16, see also Table 1). **b** Schematic illustration of positive and negative (arrows up or down) associations between hormonal and genetic risk factors and identified cells in stroma of histologically normal TDLUs

Double-labeled ALDH1+ vinculin+ s/p cells were detected in all patients (Fig. 5), often intermingled with cells that were positive solely for ALDH1 or vinculin (Fig. 5). An illustration of the frequency of observed double immunophenotypes is provided in Fig. 6. A table containing data from double immunofluorescence experiments is available in the Additional files 1, 2, 3, 4 and 5.

### Quantitative analyses of IHC Labelings with clinical correlation

**Fig. 7:**
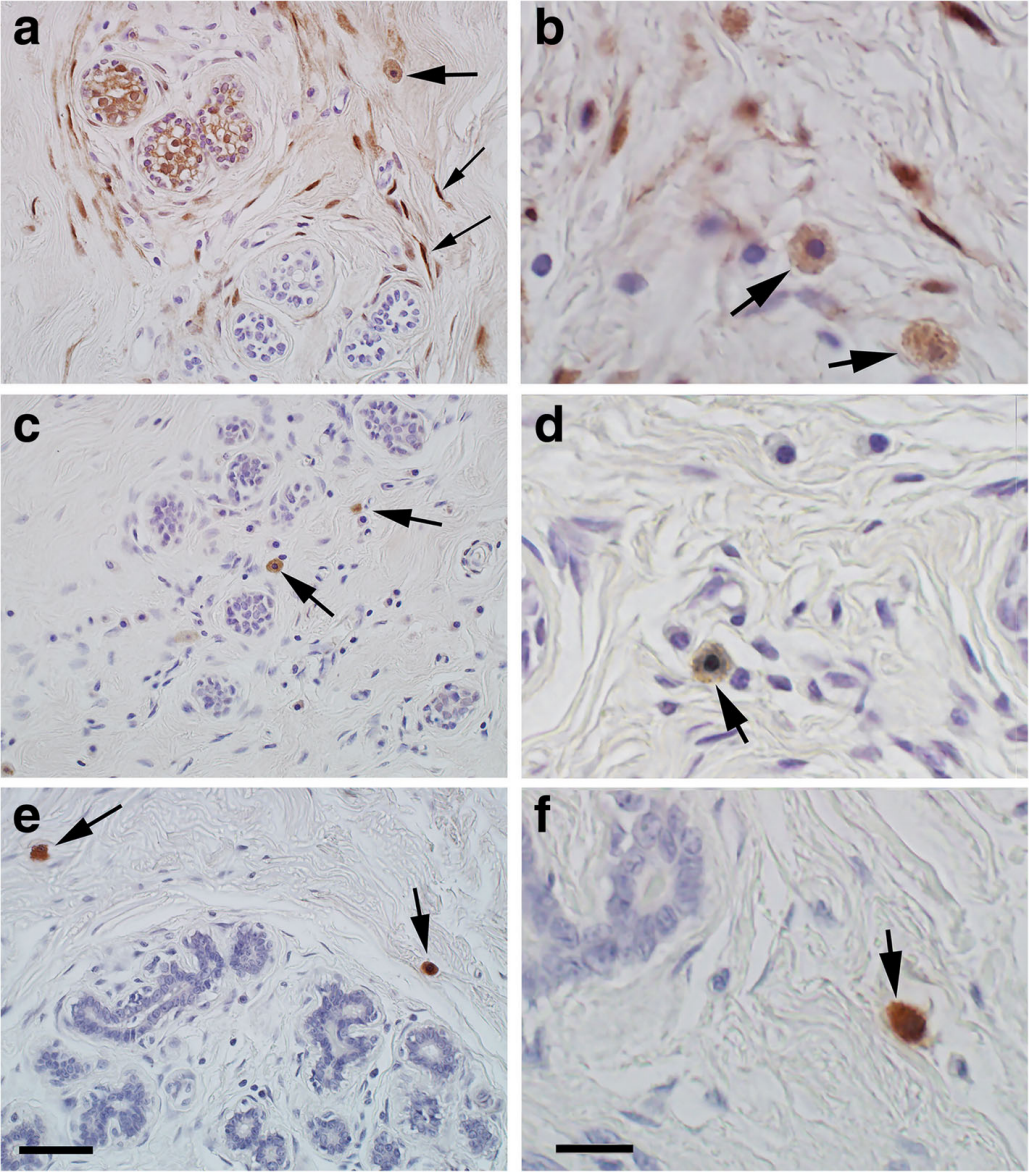
Representative images demonstrating single immunohistochemical labelings performed on morphologically normal breast specimens from 90 patients. The immunolabeling was used for quantitative assessments of ALDH1, SSEA3, and tryptase r/o and/or s/p cells that were statistically evaluated in relation to patient groups. **a** and **b** The two identified ALDH1 immunoreactive cell types: r/o (thick arrows) and s/p (thin arrows). **c** and **d** Images showing the identified r/o SSEA3+ cell type (arrows). **e** and **f** Images illustrating the identified tryptase r/o cell type (arrows). Scale bars: 50 μm in **a**, **c**, and **e**; 10 μm in **b**, **d**, and **f**

The identities of stromal r/o and s/p cells were analyzed by single IHC labeling of ALDH1, SSEA3, and tryptase. Samples from 90 patients were of adequate technical quality (tissue from 90 patients was non-analyzable) IHC single labeling was used for statistical correlations with clinical records. Representative images of the IHC labeling are shown in Fig. 7.

R/o cells were ALDH1+ in samples from 65% of the patients, and such cells were present in a median of 5% of the TDLUs. The corresponding figures for SSEA3+ r/o cells were considerably higher (99 and 43%), and for tryptase+ r/o cells still higher (99 and 67%). Significant correlations between the number of TDLUs containing the different r/o cell immunophenotypes were found for the following: SSEA3+ and tryptase+ for the whole patient set (*p* = 0.001); ALDH1+ and tryptase+ for the premenopausal group only (*p* = 0.033). In contrast, the correlation coefficient was very low for ALDH1+ and SSEA3+ r/o cells (0.168), and the *p* value indicated no statistical significance for co-presence of these two immunophenotypes in tissue samples.

Regarding ALDH1+ r/o cells in TDLU stroma, the strongest statistic significance for an association with data from clinical records was found for larger numbers of these cells with decreasing parity, and only for the patient group with *BRCA1* or *BRCA2* mutations (*p* = 0.022). Among non-carriers, parous women had higher numbers of these cells (*p* = 0.057). Furthermore, when ongoing oral contraceptive use was included in the model, a near-significant positive association was found between high ALDH1+ r/o cells and family history for premenopausal women (*p* = 0.058).

The number of SSEA3+ r/o cells associated with different risk factors for breast cancer was clearly significant. SSEA3+ r/o cells in TDLU stroma showed a positive association with a family history of breast cancer for all women (*p* = 0.021), and also for premenopausal women when the current use of oral contraceptives was included in the model (*p* = 0.009). With increasing parity, there was a significant negative association with SSEA3+ r/o cells for the total patient group (*p* = 0.015). However, when data on premenopausal patients only were analyzed and adjusted for ongoing oral contraceptive use, there was a negative association between SSEA3+ r/o cells and nulliparity (*p* = 0.042). Our results also indicated a positive association between SSEA3+ r/o cells in TDLU stroma and hormone use at the time of surgery in postmenopausal women (*p* = 0.032).

No statistically significant associations were observed between the number of tryptase+ r/o cells in TDLU stroma and risk factors for breast cancer.

ALDH1+ s/p cells were detected by chromogenic IHC in TDLU stroma of 92% of the patients (83/90) and were typically present in the majority of the TDLUs (median 75%). These cells were about half as common in the TDLUs of premenopausal patients with a family history of breast cancer compared to the TDLUs of premenopausal subjects with no family history (median 46% vs. 88%, *p* = 0.003). A reduced regression model including parity, family history, and age indicated that ALDH1+ s/p cells were negatively associated with family history both for all women (*p* = 0.001) and for premenopausal women (*p* = 0.001). This association was not significant when ongoing hormone use were included in the model.

No SSEA3 or tryptase labeled s/p cells were detected. The positive and negative associations identified between cell types and examined risk factors are illustrated in Fig. 6b.

## Discussion

### Round/oval stromal cells

R/o cells in stroma were previously shown to be negative for Cam5.2 in this patient material, thus ruling out epithelial cell type [14]. In histologically normal TDLUs, co-expression in r/o cells was revealed for all combinations of double-labeling of ALDH1, SSEA3, and tryptase in the vast majority of the specimens from the investigated patients. Of these three epitopes, only tryptase is considered to be a marker of mast cell identity, although we noted that ALDH1+ r/o cells and SSEA3+ r/o cells also exhibited mast-like morphology, including degranulation. These findings, together with significant co-presence of tryptase+ cells and SSEA3+ cells, and co-presence of tryptase+ cells and ALDH1+ cells, suggests that these cell types either are, or have functions in common with, mast cells. However, there was no correlation between the presence of ALDH1+ r/o cells and SSEA3+ r/o cells in the investigate material. These two r/o immunophenotypes may thus represent different cells, an interpretation that is further supported by the demonstrated differences in associations with risk factors between these two immunotypes. Furthermore, tryptase+ r/o cells were not statistically associated with any of the examined risk factors, which indicates that differentiated mast cells do not play a role in the risk of breast cancer.

We have previously shown that the granular cytoplasm of ALDH1+ r/o cells strongly expresses the contractile protein marker SMMHC [14], which is compatible with a migratory quality that can be expected in bone-marrow-derived cells. Importantly, the r/o cellular morphology found in the above-mentioned immunophenotypes is congruent with the pluripotent mesenchymal cells called multilineage stress-enduring (Muse) cells, with the immunophenotypic identity defined by SSEA3 positivity [21, 22]. For in situ studies, which include morphologic assessment of cell location, it is not necessary to apply the other determinant of Muse cells, the mesenchymal cell marker CD105 [21, 22]. The pluripotency of SSEA3+ cells has been confirmed in goats [28], and SSEA3+ cells in colorectal mucosa have been localized to stroma but not to epithelium [29]. In contrast, two studies have reported that SSEA3+ stromal cells are absent in breast tissue [12, 30]. However, the mentioned observations in mammary tissue were based solely on flow cytometry (i.e., dependent on cell surface immunolabeling), and hence they do not conflict with our results or the findings outlined by Dezawa et al. [22] in which IHC demonstrated that SSEA3 is present in the cytoplasm.

According to our data, ALDH1+ r/o cells are negatively associated with parity in *BRCA1/2* mutation carriers but positively associated with parity in non-carriers. This is very interesting, because different numbers of pregnancies have been reported to have opposing effects on the risk of breast cancer in women carrying the *BRCA* mutation [31–33].

We detected an increased presence of ALDH1+ r/o cells in premenopausal women with family history of breast cancer, this was statistically near-significant and was found only after adjusting for oral contraceptives.

Our previous investigation of benign mammary stroma indicated that low presence of ALDH1+ CD44+ CD24– r/o cells in premenopausal women is marginally correlated with a family history of breast cancer, but that this does not apply to ALDH1+ CD44– CD24– cells [20]. Although findings in the current study were not statistically significant for ALDH1+ r/o cells, they do suggest that the presence of such cells in histologically normal TDLUs is affected by hormones.

In the current study we found that SSEA3+ r/o cells were significantly associated with family history when all patients were analyzed together, and this association was even stronger when premenopausal patients were investigated separately. SSEA3+ r/o cells were also associated with low parity, and a weaker negative association was noted for the premenopausal group but only when the data were adjusted for ongoing use of oral contraceptives. These apparently menopause- and contraceptive pill-dependent differences in associations between SSEA3+ r/o cells and parity strongly indicate a hormonal link and merit further investigation.

We observed a signifiant relationship between SSEA3+ r/o cells and ongoing postmenopausal hormone use, which suggests that such treatment increases the number of mesenchymal stem cells in breast tissue. Notably, other investigators have reported more extensive stroma in hormone-treated patients [34].

Our morphologic and immunohistologic findings for SSEA3+ cells correspond to the above-mentioned Muse cells [12, 21, 22], which supports the assumption that the r/o stromal cell population in benign breast stroma contains mesenchymal stem cells. It is at best premature to suggest a causative mechanism between SSEA3+ r/o cells and breast cancer. Muse cells are derived from bone marrow, have powerful tissue regeneration properties [35], and increase markedly in number in circulating blood after ischemic injury [36]. Accordingly, large numbers of SSEA3+ r/o cells may reflect a microenvironment that is responding to tissue injury, which would be congruent with early oncogenesis.

### Spindle-shaped/polygonal cells

The present study aimed to test the hypothesis that the ALDH1-expressing s/p cells in TDLU stroma are stellate cells, a term that refers to a specific cell type (also known as vitamin A-storing cell) [24, 26]. In all of the investigated patients s/p cells in the TDLU stroma co-labeled for ALDH1 and the stellate cell marker vinculin, and groups of s/p cells exhibited variations in positivity for either or both those markers. Other researchers have reported that vinculin is efficacious in labeling resting stellate cells [37]. Thus the morphology and the immunophenotype of the s/p cells identified in the current study are entirely consistent with stellate cells. Due to the close relationship between ALDH1 and vinculin expression in s/p cells, IHC to assess clinical correlation was performed only for ALDH1 in our investigation. The results indicated a highly significant association between low numbers of ALDH1+ s/p cells in premenopausal women and family history of breast cancer. Correspondingly, in our recent study of mammary stroma in premenopausal women [20], low occurrence of ALDH1+ s/p cells was associated with the risk factor nulliparity.

These data linking relative lack of ALDH1+ s/p cells with breast cancer risk factors should be viewed considering that ALDH1 is an enzyme that catalyzes the production of retinoic acid (the physiological end product of vitamin A), a compound necessary for cell differentiation. It is logical to argue that lack of drivers of epithelial cell differentiation may predispose to carcinoma. In keeping with this, a recent study indicated that expression of ALDH1 in breast tumor stroma is associated with favorable outcome [18], and retinoic acid alone can trigger the differentiation of malignant cells and cause clinical remission [38].

Only a few studies have evaluated stromal cell types in normal breast tissue. One investigation distinguished two different types of elongated stromal cells with identical morphology, one present in TDLUs and the other found in generic stroma of the breast [39]. The former cell type was characterized as having a CD105^high^ and CD26^low^ immunophenotype, and only this population stimulated epithelial growth and branching from epithelial progenitor cells. The authors described this cell type as fibroblasts (i.e. morphology similar to that of stellate cells), and thus it is possible that we are describing the same cells in the present study. Regardless of whether that is the case, future research should focus on determining whether retinoic acid deficiency in subepithelial stroma is a contributing factor in mammary carcinogenesis.

## Conclusion

This study describes immunohistologic characteristics of several cell types that are novel for benign human female breast tissue. According to clinical data presented here these cells are associated with genetic and hormonal risk factors for breast cancer.

## Additional files

Additional file 1: Methodologic considerations. (DOCX 102 kb)

Additional file 2: **Table S1.** Results from double immunofluorescence experiments. (DOCX 42 kb)

Additional file 3: **Table S2.** Single immunohistochemistry data. Sortable by cell location and morphology, and by reduced clinical data. (XLSX 15 kb)

Additional file 4: Key for column names and coded content in single immunohistochemistry data table. (DOCX 13 kb)

Additional file 5: Supplementary Figure A representative dIF image of a tissue section analyzed in this study, here without primary antibody, demonstrating absence of nonspecific binding or background signals. Blue: histochemical nuclear staining by DAPI. (JPEG 1660 kb)

## Abbreviations

ALDH1: Aldehyde dehydrogenase 1 A1
dIF: double immunofluorescence
FFPE: Formalin-fixed, paraffin-embedded
H&E: Hematoxylin and eosin
IHC: Immunohistochemistry
R/o cells: Round or oval cells
S/p cells: Spindle-shaped or polygonal cells
SMMHC: Smooth muscle myosin heavy chain
SSEA3: Stage-specific embryonic antigen-3
TDLU: Terminal duct-lobular unit
