# Supplementary material for: Stellate cells and mesenchymal stem cells in benign mammary stroma are associated with risk factors for breast cancer – an observational study
Source: BMC Cancer. 2018 Feb 27;18:230. doi: 10.1186/s12885-018-4151-x (PMC6389039; doi:10.1186/s12885-018-4151-x)
Supplement: Supplementary file 1 — Methodologic considerations. (DOCX 102 kb) [file 12885_2018_4151_MOESM1_ESM.docx]

**Methodologic Considerations**

The experimental approach applied in this study used IHC techniques that could visualize cells *in* situ to reveal their morphology and spatial relationships with neighboring structures. Cell identification was based on assessment of cell location and morphology (including shape and cytoplasmic features), together with visualization of immunolabeled proteins that characterize specific cell identities according to previous scientific reports. Cell-specific immunolabeling was ensured by using appropriate positive and negative control tissue and antibody specificity controls of labeling and labeling quality, and by performing confocal microscopy to determine possible cellular co-localization.

The most "normal" patient subgroup in this study comprised non-cancer and non-carrier women who were in need of mammoplasty, and hence these subjects were not entirely free from breast disorders. However, whole tissue sections from living women without any breast disease or genetic predisposition are rare. We preferred using full-size tissue sections, because some of the cell types we investigated are sparsely distributed. The material from each patient comprised tissue with a median area of 255 (range 48–621) sq mm, which is about 900 times larger than a single tissue microarray sample. It should also be noted that the tissue originated from FFPE blocks that had been archived for up to 33 years. This admittedly led to quality problems with the oldest specimens in terms of friability and poor adhesion to microscope slides, which resulted in rejection of up to 10% of specimens from some analysis series due to less than 10 TDLUs being represented on IHC slides.

As expected, immunoreactivity was detected with greater sensitivity by dIF experiments than by IHC labeling. This was most evident regarding ALDH1+ r/o cells, which were documented in the TDLU stroma of all patients in the subset investigated by dIF, but in only 65% of the total patient material when using IHC analysis. Also, considering the different microscopy approaches, we noted that confocal microscopy offered higher sensitivity for double-positive cells compared to epifluorescence microscopy due to better visual separation of signals. In general, this is not considered to reduce the validity of chromogenic IHC evaluation to detect differences in the occurrence of specific cell types between clinical subgroups.
