# Supplementary material for: Stellate cells and mesenchymal stem cells in benign mammary stroma are associated with risk factors for breast cancer – an observational study
Source: BMC Cancer. 2018 Feb 27;18:230. doi: 10.1186/s12885-018-4151-x (PMC6389039; doi:10.1186/s12885-018-4151-x)
Supplement: Supplementary file 2 — Table S1. Results from double immunofluorescence experiments. (DOCX 42 kb) [file 12885_2018_4151_MOESM2_ESM.docx]

**Table S1**

**Results from double immunofluorescence experiments** using morphologically normal breast tissue from 16 patients randomly selected from subgroups according to hormonal and genetic risk factors; same patients in same order as described in Table 1. All were of age 31-40 years. This table indicates presence (yes) or absence (no) of cells with each immunophenotype in the specimen from each examined patient. Dashes indicate that the feature could not be interpreted due to suboptimal technical specimen quality.

Abbreviations: R/o, round/oval cells. S/p, spindle-shaped or polygonal cells.

**A. Tryptase and SSEA3**

|  | Tryptase+ SSEA3+ | | | Tryptase+ SSEA3- | | | Tryptase- SSEA3+ | | |
| --- | --- | --- | --- | --- | --- | --- | --- | --- | --- |
|  | Stroma | | Ductule | Stroma | | Ductule | Stroma | | Ductule |
| Patient | R/o | S/p |  | R/o | S/p |  | R/o | S/p |  |
| 17 | Yes | No | No | Yes | No | No | Yes | No | No |
| 75 | Yes | No | No | Yes | No | No | Yes | No | No |
| 91 | Yes | No | No | Yes | No | No | Yes | No | No |
| 62 | Yes | No | No | Yes | No | No | Yes | No | No |
| 97 | Yes | No | No | Yes | No | No | Yes | No | Yes |
| 59 | Yes | No | No | Yes | No | No | Yes | No | No |
| 89 | Yes | No | No | Yes | No | No | Yes | No | No |
| 78 | Yes | No | No | Yes | No | No | Yes | No | No |
| 30 | No | No | No | Yes | No | No | Yes | No | No |
| 83 | Yes | No | No | Yes | No | No | Yes | No | No |
| 46 | Yes | No | No | No | No | No | Yes | No | No |
| 99 | Yes | No | No | Yes | No | No | Yes | No | No |
| 103 | Yes | No | No | Yes | No | No | Yes | No | No |
| 116 | Yes | No | No | Yes | No | No | Yes | No | No |
| 37 | Yes | No | No | Yes | No | No | No | No | No |
| 126 | Yes | No | No | Yes | No | No | Yes | No | Yes |

**B. ALDH1 and vinculin**

|  | ALDH+ vinculin+ | | | ALDH+ vinculin- | | | ALDH- vinculin+ | | |
| --- | --- | --- | --- | --- | --- | --- | --- | --- | --- |
|  | Stroma | | Ductule | Stroma | | Ductule | Stroma | | Ductule |
| Patient | R/o | S/p |  | R/o | S/p |  | R/o | S/p |  |
| 17 | No | Yes | No | Yes | Yes | No | No | Yes | No |
| 75 | Yes | Yes | No | Yes | Yes | No | Yes | Yes | Yes |
| 91 | Yes | Yes | No | No | Yes | No | Yes | Yes | Yes |
| 62 | Yes | Yes | Yes | No | Yes | No | Yes | Yes | Yes |
| 97 | Yes | Yes | Yes | Yes | Yes | No | Yes | Yes | Yes |
| 59 | Yes | Yes | Yes | Yes | Yes | No | Yes | Yes | Yes |
| 89 | Yes | Yes | No | Yes | Yes | No | Yes | Yes | Yes |
| 78 | Yes | Yes | Yes | Yes | Yes | No | Yes | Yes | Yes |
| 30 | Yes | Yes | — | Yes | Yes | — | No | Yes | — |
| 83 | Yes | Yes | Yes | Yes | Yes | No | Yes | Yes | Yes |
| 46 | Yes | Yes | Yes | No | Yes | No | Yes | Yes | No |
| 99 | Yes | Yes | No | Yes | Yes | No | Yes | Yes | Yes |
| 103 | Yes | Yes | Yes | No | Yes | No | Yes | Yes | Yes |
| 116 | Yes | Yes | — | No | Yes | — | Yes | Yes | Yes |
| 37 | — | — | — | — | — | — | — | — | — |
| 126 | No | Yes | Yes | No | Yes | No | Yes | No | Yes |

**C. ALDH1 and SSEA3**

|  | ALDH1+ SSEA3+ | | | ALDH1+ SSEA3- | | | ALDH1- SSEA3+ | | |
| --- | --- | --- | --- | --- | --- | --- | --- | --- | --- |
|  | Stroma | | Ductule | Stroma | | Ductule | Stroma | | Ductule |
| Patient | R/o | S/p |  | R/o | S/p |  | R/o | S/p |  |
| 17 | Yes | No | No | No | Yes | No | Yes | No | No |
| 75 | No | No | No | Yes | Yes | No | Yes | No | No |
| 91 | Yes | No | No | Yes | Yes | No | Yes | No | No |
| 62 | Yes | No | No | Yes | Yes | No | Yes | No | No |
| 97 | Yes | No | No | Yes | Yes | No | Yes | No | No |
| 59 | Yes | No | No | Yes | Yes | No | Yes | No | No |
| 89 | Yes | No | No | Yes | Yes | No | 0 | No | No |
| 78 | Yes | No | No | Yes | Yes | No | Yes | No | Yes |
| 30 | Yes | No | No | No | Yes | No | Yes | No | No |
| 83 | Yes | No | No | No | Yes | No | Yes | No | No |
| 46 | No | No | No | No | Yes | No | Yes | No | No |
| 99 | Yes | No | No | Yes | Yes | No | Yes | No | No |
| 103 | Yes | No | No | Yes | Yes | No | Yes | No | No |
| 116 | No | No | No | Yes | Yes | No | Yes | No | No |
| 37 | Yes | No | No | No | Yes | No | 0 | No | No |
| 126 | Yes | No | No | Yes | Yes | No | Yes | No | No |

**D. ALDH1 and tryptase**

|  | ALDH+ tryptase+ | | | ALDH+ tryptase- | | | ALDH- tryptase+ | | |
| --- | --- | --- | --- | --- | --- | --- | --- | --- | --- |
|  | Stroma | | Ductule | Stroma | | Ductule | Stroma | | Ductule |
| Patient | R/o | S/p |  | R/o | S/p |  | R/o | S/p |  |
| 17 | No | No | No | Yes | Yes | No | Yes | No | No |
| 75 | Yes | No | No | Yes | Yes | No | Yes | No | No |
| 91 | Yes | No | No | Yes | Yes | Yes | Yes | No | No |
| 62 | Yes | No | No | Yes | Yes | No | Yes | No | No |
| 97 | Yes | No | No | Yes | Yes | Yes | Yes | No | No |
| 59 | Yes | No | No | No | Yes | No | Yes | No | No |
| 89 | Yes | No | No | No | Yes | No | Yes | No | No |
| 78 | Yes | No | No | No | Yes | No | Yes | No | No |
| 30 | Yes | No | No | Yes | Yes | No | Yes | No | No |
| 83 | Yes | No | No | Yes | Yes | No | Yes | No | No |
| 46 | Yes | No | No | No | Yes | No | Yes | No | No |
| 99 | Yes | No | No | No | Yes | No | Yes | No | No |
| 103 | Yes | No | No | No | Yes | No | Yes | No | No |
| 116 | Yes | No | No | Yes | Yes | No | Yes | No | No |
| 37 | Yes | No | No | No | Yes | No | Yes | No | No |
| 126 | Yes | No | No | Yes | Yes | No | Yes | No | No |
