# Supplementary material for: Stellate cells and mesenchymal stem cells in benign mammary stroma are associated with risk factors for breast cancer – an observational study
Source: BMC Cancer. 2018 Feb 27;18:230. doi: 10.1186/s12885-018-4151-x (PMC6389039; doi:10.1186/s12885-018-4151-x)
Supplement: Supplementary file 4 — Key for column names and coded content in single immunohistochemistry data table. (DOCX 13 kb) [file 12885_2018_4151_MOESM4_ESM.docx]

**Key for column names, data table 2016-12-08**

0=no; 1=yes.

BRCA column: 1=BRCA1 mutation; 2= BRCA2 mutation.

| **Case** | Case number |
| --- | --- |
| ***Duct_ALDH*** | Ductules, fraction of TDLUs with one ore more ALDH1+ cells present |
| ***SP_ALDH*** | TDLU stroma, fraction of TDLUs with one ore more ALDH1+ spindle-shaped/polygonal cells present |
| ***RO_ALDH*** | TDLU stroma, fraction of TDLUs with one or more ALDH1+ round/oval cells present |
| ***RO_Trypt*** | TDLU stroma, fraction of TDLUs with one or more Tryptase+ round/oval cells present |
| ***RO_SSEA*** | TDLU stroma, fraction of TDLUs with one or more SSEA3+ round/oval cells present |
| **Menop** | Menopause at time of operation |
| **Parity** | Childbirth(s) before operation |
| **Fam_hist** | Family history of breast cancer, 1^st^ grade relative |
| **BRCA** | BRCA mutation 1 or 2 |
