# Supplementary figures and images for: Stellate cells and mesenchymal stem cells in benign mammary stroma are associated with risk factors for breast cancer – an observational study
Source: BMC Cancer. 2018 Feb 27;18:230. doi: 10.1186/s12885-018-4151-x (PMC6389039; doi:10.1186/s12885-018-4151-x)

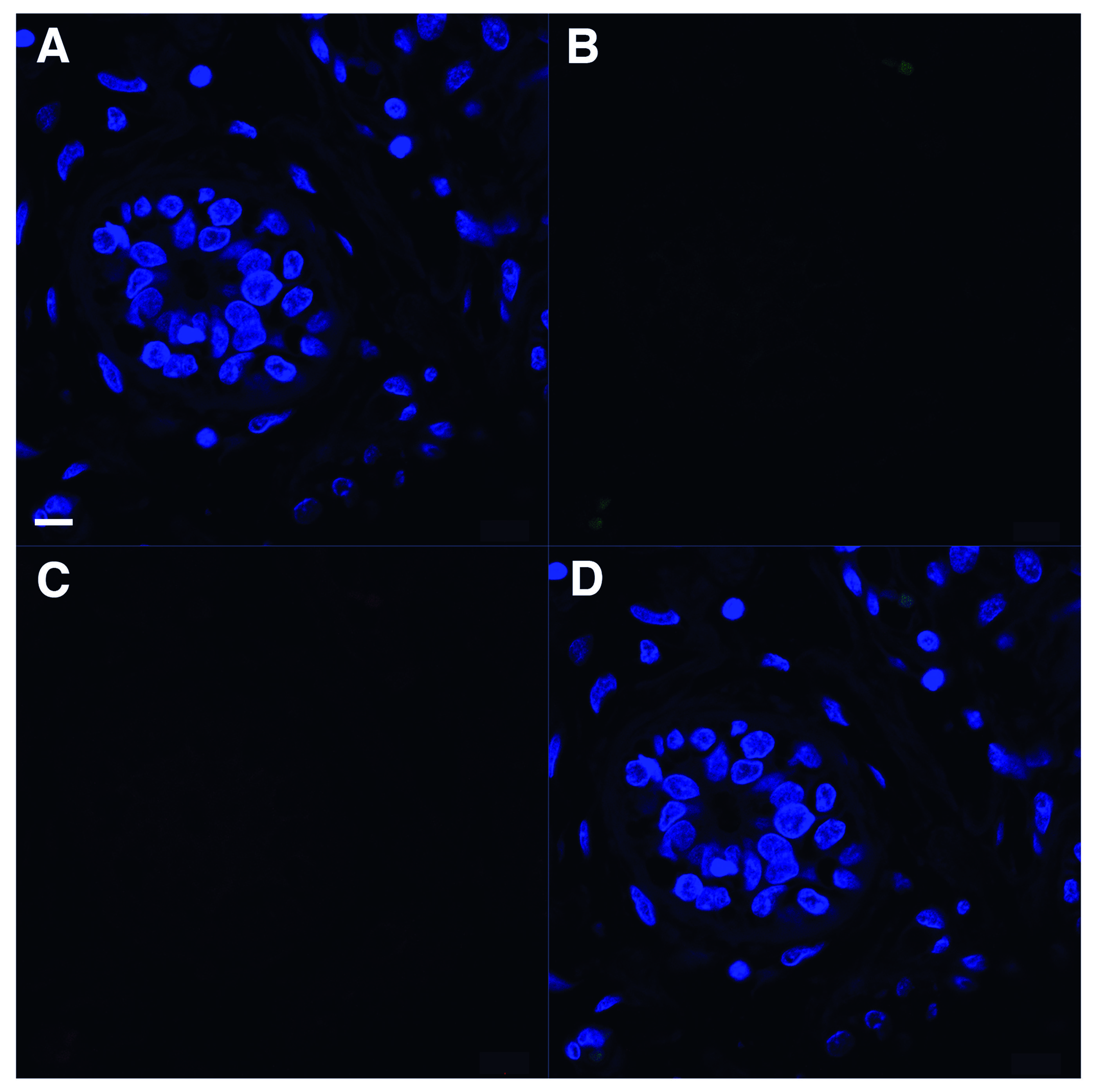

Supplement: Supplementary file 5 — Supplementary Figure A representative dIF image of a tissue section analyzed in this study, here without primary antibody, demonstrating absence of nonspecific binding or background signals. Blue: histochemical nuclear staining by DAPI. (JPEG 1660 kb) [file 12885_2018_4151_MOESM5_ESM.jpg]
